# Goal-directed and habitual control: from circuits and functions to exercise-induced neuroplasticity targets for the treatment of Parkinson’s disease

**DOI:** 10.3389/fneur.2023.1254447

**Published:** 2023-10-10

**Authors:** Talifu Zikereya, Kaixuan Shi, Wei Chen

**Affiliations:** ^1^Department of Physical Education, China University of Geosciences, Beijing, China; ^2^Physical Education College, Hebei Normal University, Shijiazhuang, Hebei, China

**Keywords:** exercise, Parkinson’s disease, goal-directed and habitual control circuits, neuroplasticity, dopamine

## Abstract

Parkinson’s disease (PD) is a neurodegenerative disease characterized by motor and cognitive impairments. The progressive depletion of dopamine (DA) is the pathological basis of dysfunctional goal-directed and habitual control circuits in the basal ganglia. Exercise-induced neuroplasticity could delay disease progression by improving motor and cognitive performance in patients with PD. This paper reviews the research progress on the motor-cognitive basal ganglia circuit and summarizes the current hypotheses for explaining exercise intervention on rehabilitation in PD. Studies on exercise mediated mechanisms will contribute to the understanding of networks that regulate goal-directed and habitual behaviors and deficits in PD, facilitating the development of strategies for treatment of PD.

## 1. Introduction

Parkinson’s disease (PD) is characterized by the fundamental pathological manifestation of a gradual decline in the ascending dopaminergic projection within the basal ganglia (1). Research conducted on animals and patients with PD has revealed spatially segregated functional regions within the basal ganglia that oversee the regulation of goal-directed and habitual actions. In individuals affected by PD, the decline in dopamine (DA) is primarily observed in the posterior putamen, a section of the basal ganglia connected to the regulation of habitual behavior (2). As a result, these patients might be compelled to increasingly rely on the goal-directed mode of action control, which is facilitated by the remaining DA in the striatum (3). Thus, many of the challenges they experience in their behavior might stem from the diminished ability to exert normal automatic control due to distorted output signals originating from habitual control circuits. These signals interfere with the performance of goal-directed action.

Rehabilitation is considered a promising treatment option in addition to surgery and medication for managing PD (4). Rehabilitation has the potential to enhance the effectiveness of drugs and minimize secondary injuries, thereby slowing the progression of PD (5). Exercise and physical activity are known to complement dopamine replacement therapy. Numerous studies have shown that aerobic exercise had beneficial effects in improving balance, gait, and motor function in PD patients (6). Furthermore, research on the mechanism of exercise-induced neuroplasticity has significantly contributed to the support and promotion of exercise rehabilitation for preventing and treating PD. Exercise interventions can enhance dopaminergic neurotransmission in the nigrostriatal pathway and attenuate overexcitable glutamatergic transmission in the corticostriatal pathway (7). While studies support the importance of exercise and physical activities to achieve motor improvements, the exact mechanisms underlying these effects remain unclear, which ultimately hinders the development of exercise treatment strategies for patients with PD. With the aim of promoting the development of PD treatment strategies in clinical practice and improving the existing understanding of pathological mechanisms, this article focuses on the dual system of goal-directed behavior and habitual control. This article reviews the role of the dual system in the occurrence and development of PD as well as the neuroplastic mechanisms of exercise in improving PD.

### 1.1. Anatomy of the basal ganglia: the classical model of direct and indirect pathways

The basal ganglia is a group of subcortical nuclei mainly involved in skill learning and motor control. The striatum is the main input unit of the basal ganglia, and the medium spiny neurons (MSNs), which account for approximately 95% of the striatal neurons, receive excitatory glutamatergic inputs from the cerebral cortex and dopaminergic regulation from the substantia nigra pars compacta. Simultaneously, the MSNs project γ-aminobutyric acid outputs into the downstream nuclei. MSNs are categorized into two types according to their expressed receptors: (1) MSNs expressing type 1 dopamine receptors (D1-MSNs) project fibers to the substantia nigra pars reticulate and the globus pallidus internus (GPi), constituting the direct pathway; (2) MSNs expressing type 2 dopamine receptors (D2-MSNs) project fibers to the globus pallidus externus (GPe), which together with the GPe-subthalamic nucleus (STN)-substantia nigra pars reticulate/GPi projections, constituting the indirect pathway (8).

**Figure 1:**
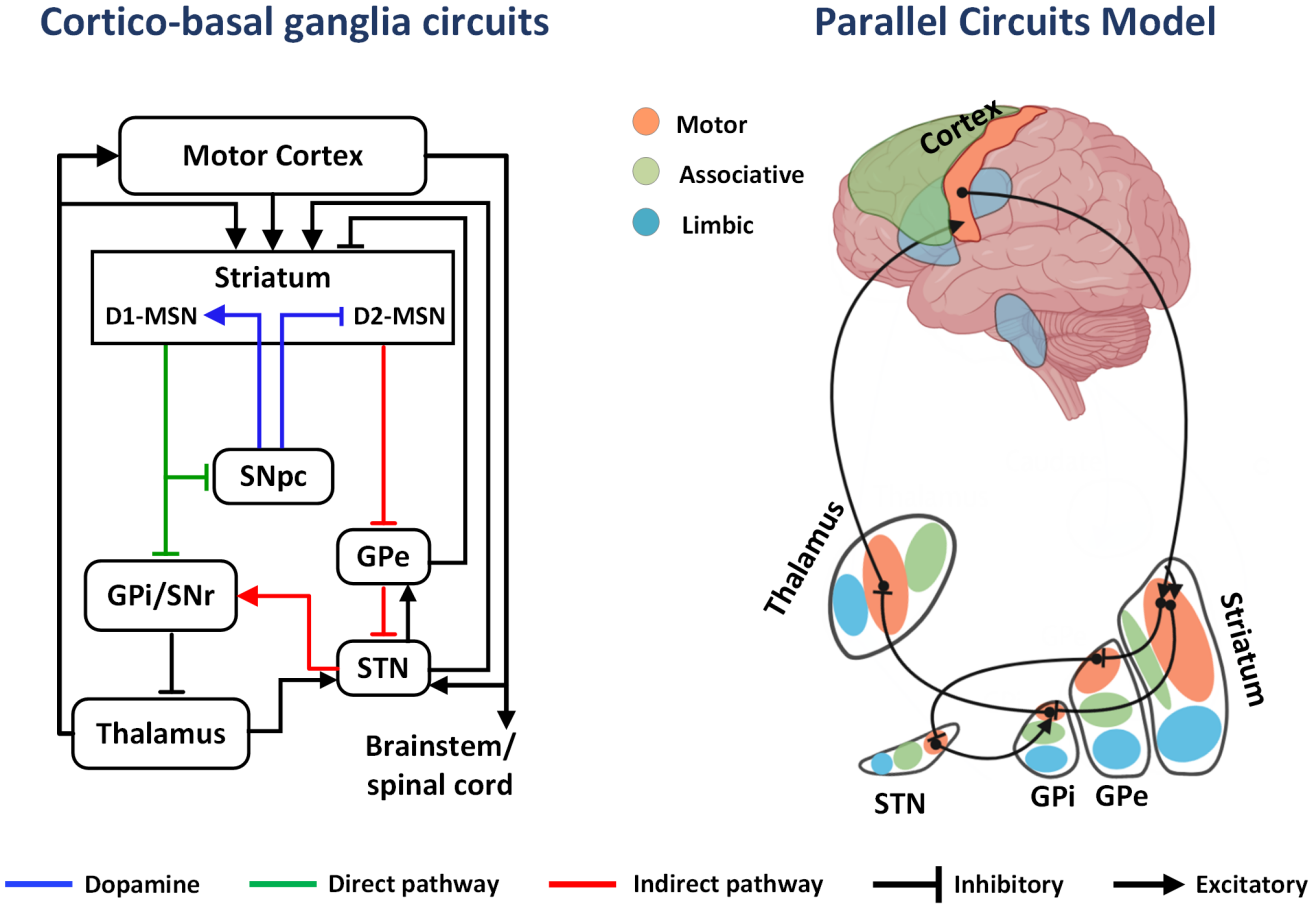
The cortico-basal ganglia circuit and parallel model circuits. In the basal ganglia circuit, DA from the SNpc to the striatum activates direct pathway (D1-MSNs) and inhibits indirect pathway MSNs (D2-MSNs). This effect decreases GPi output, releasing inhibition on the thalamus and cortex and promoting movement. Limbic, associative, and sensorimotor information from excitatory cortical afferents is distributed in parallel to regions of the striatum. As an example, inhibitory projections from sensorimotor striatum innervate sensorimotor regions of the GPi either directly or intermediately through the GPe and STN. Projections from sensorimotor regions of GPi then innervate the motor thalamus, which projects back to sensorimotor regions of the cortex.

**Figure 2:**
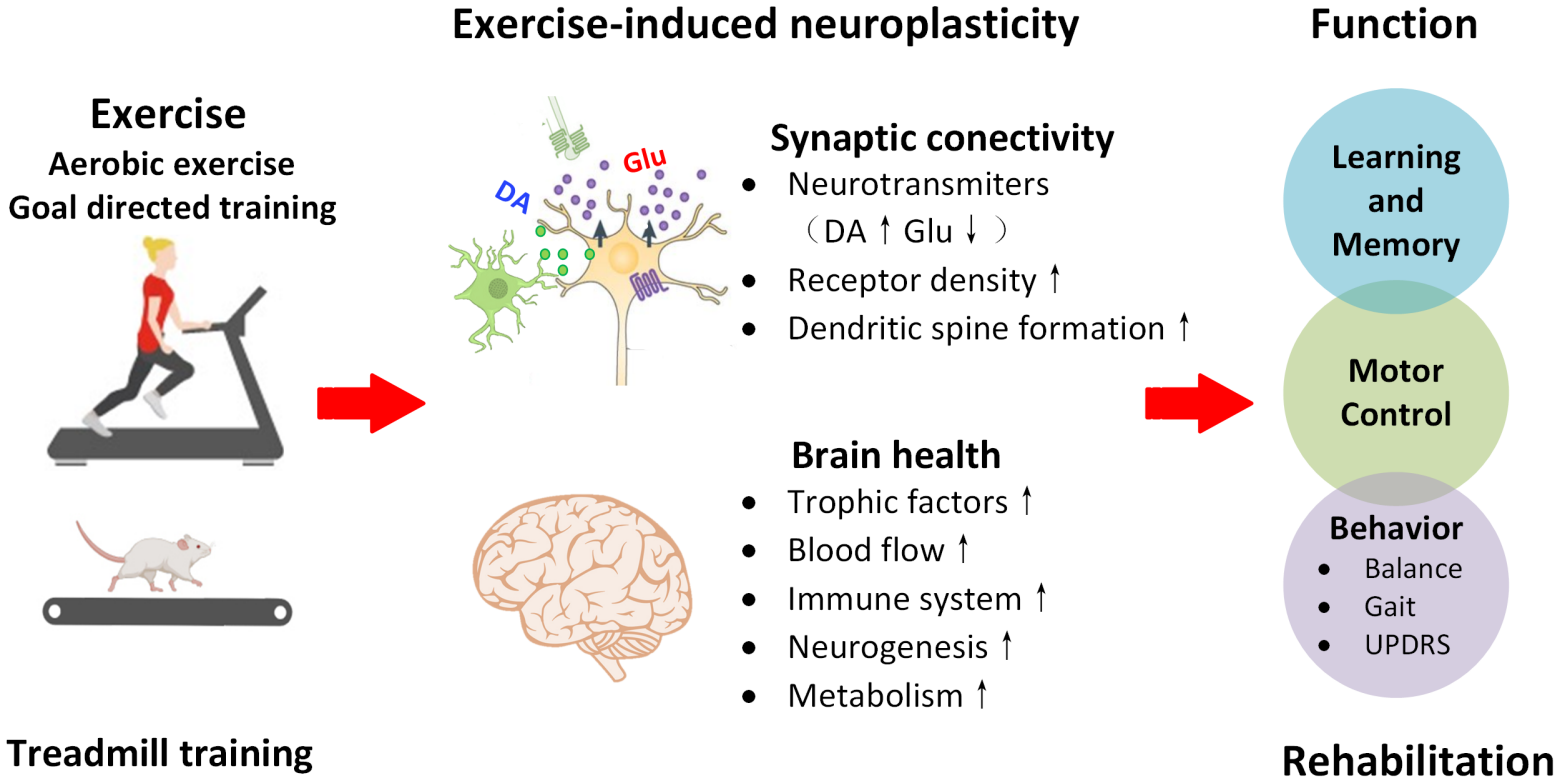
Exercise mediated neuroplasticity. Both aerobic exercise and goal-directed exercise can reduce the effects of oxidative stress, promote excitation-inhibition balance of DA-Glu neurotransmitters, and enhance synaptic structural and functional plasticity by regulating neurotransmitter conduction in DA and non-DA systems. In addition, exercise improves brain health by promoting the expression of neurotrophic and anti-inflammatory factors. Exercise mediated neuroplasticity is the neural basis of PD motor control, learning and memory, and improved behavioral performance.

The classical model of direct and indirect pathways was first proposed by Albin (9). According to this model, activation of the direct pathway facilitates movement, while the indirect pathway is responsible for behavioral suppression. The proposal of this model led to further clinical and animal research, but the subsequent studies have highlighted the limitations of the theory in explaining the mechanisms related to motor control and skill learning. Anatomical evidence yielded the following findings: (1) D1-MSNs project some fibers to the GPe; (2) in the indirect pathway, GPe neurons project to the STN and also project some fibers to substantia nigra pars reticulate/GPi; (3) the STN receives projections from the cortex and multiple nuclei and simultaneously projects fibers to the GPe to form synaptic connections (10). The complex structural connections break the top-down feedforward model of the original indirect pathway (Figure 1). Alexander et al. (11) proposed a parallel circuit model for the basal ganglia network in 1986, which divided the striatum into three functional areas: the ventral striatum, the caudate nucleus, and the putamen. The parallel model considers direct and indirect pathways as subsets of the cortico-basal ganglia circuit, which shows highly differentiated functional characteristics. With advancements in electrophysiology and brain imaging techniques, the basal ganglia circuits have now been divided into three functional loops, namely, the limbic, associative, and sensorimotor circuits, which are responsible for execution and regulation of motor control, learning, rewards, and motivation (Figure 2).

## 2. Functional organization of the basal ganglia: the goal-directed and habitual control circuit model

Motor learning is a complex process that involves both explicit and implicit learning. Explicit and implicit learning refers to the process by which memories are stored. Explicit learning is a conscious and intentional process, while implicit learning is unconscious and incidental (12). Both the explicit (spared) and the implicit (deficient) learning modalities are utilized in PD, and this represents a great cognitive advantage for motor rehabilitation (4). Researchers have successfully mapped three basal ganglia functional networks based on the joint learning model, which involve both goal-directed and habitual control systems (13). Goal-directed behavior acquisition is a dynamic process of in-depth processing of the association between behavioral responses and potential consequences. This process can adjust behavioral responses according to changes in the value of the consequences with a high degree of flexibility, but it consumes relatively more cognitive resources compared to automatic behavior. Habitual behavior learning, also known as stimulus–response associations, involves the reinforcement of existing stimulus–response associations. Habitual behavior learning is characterized by automation and efficiency but also inflexibility. Previous studies have improved the theory of the dual-system control model, and researchers have proposed a variety of models of competition and cooperation between the two (14, 15).

The basal ganglia is also involved in the integration and selection of voluntary behavior. The striatum is the main input station for the basal ganglia and plays a key role in instrumental behavior (learned behavior that is modified by consequences) (16). Therefore, the pathways connecting the basal ganglia, cortex, and cerebellum not only plan specific actions as sequences of movements, but also influence the execution of movements by incorporating emotions, motivational factors, and cognitive processes that organize movement strategies. The elements that facilitate motor behaviors are evident in the anatomical and functional arrangement between the basal ganglia and its projections to cortical regions, particularly the sensorimotor cortex (17). This cortex plays a vital role in processing sensory inputs that are essential for skilled movement. Furthermore, the prefrontal cortex (PFC), involved in cognition, emotions, and motivation, also projects to the ventral striatum and rostral caudate nucleus, complementing the caudal projections primarily from sensorimotor cortical areas to the putamen (18).

### 2.1. Cooperation and competition between the goal-directed and habitual control systems

The research on goal-directed and habitual control systems originates from the stimulus–response theory and cognitive map theory in Psychology (19). According to the stimulus–response theory, with an increase in training, the time for different species to reach the target result is shortened, the error rate is reduced, and the stimulus–response association is strengthened, which leads to behavior acquisition (20). On the other hand, the cognitive map theory suggests that individual behavioral learning is based on the response to environmental cognition (19). The collision of these two theories has motivated initial studies of the goal-directed and habitual control systems. In the initial stage of stimulus–response theory, researchers designed many intricate animal experiments based on research paradigms that adequately determined whether the behavior was goal-directed or habitually controlled. However, with advancements in computational science and machine learning methods, researchers believe that both elements of the dual system can cooperate closely and switch flexibly in real time when individuals make behavioral responses to the external environment and have established corresponding models based on the reinforcement theory. Balleine et al. (21) found that many skills can be broken down into sequential combinations regulated by both goal-directed and habitual control systems. Daw et al. (22) adopted algorithms to explain the tandem working mechanism of the dual system and found that the loop structures connecting the cortex and the relevant functional regions of the basal ganglia are likely to play a key role in the switch between habitual and goal-directed behavioral control modes.

Patients with PD who experience dyskinesia exhibit various degrees of impairment in dopaminergic neurons and striatal dopamine content within the substantia nigra pars compacta (SNpc). Positron emission tomography scans have revealed that the primary reduction in dopamine occurs in the tail region of the striatum (23). This observation corresponds with post-mortem data, which demonstrates the most significant loss of dopaminergic innervation in the posterior putamen, accompanied by a corresponding decline in dopaminergic cells in the ventrolateral SNc. Hence, dysfunction in the sensorimotor circuits of the basal ganglia indicates the initial stages of PD. Animal studies have also provided evidence of an imbalance in the dual system. Even after extensive training, rats with PD continue to display goal-oriented control behavior while struggling to establish habitual behavior (24).

Both goal-directed and habituation control circuits transmit parallel signals to downstream brain regions, collectively regulating movement. Following striatal denervation, the signals from the goal-directed systems must counteract the robust inhibition from the habituation control systems to effectively execute actions. Abnormal signal transmission in the basal ganglia of individuals with PD and models of movement abnormalities can be observed, manifested by an increased firing frequency of ganglia and abnormal synchronized oscillatory patterns in the subthalamic nucleus (STN) and globus pallidus interna (GPi) (25, 26). Imaging investigations in patients with PD have revealed both heightened and diminished activity in the sensorimotor cortex-dorsal striatum (SMC-DMS) pathway. During the second phase of skill acquisition, the functional connectivity of the prefrontal cortex-dorsolateral striatum (PFC-DLS) pathway does not exhibit a reduction, which implies that neural control does not shift from the goal-oriented pathway to the habit-based pathway (17). These findings suggest that the goal-directed control system must overcome the pathological signals originating from the habituation control system in order to execute the original habitual exercise. This may elucidate various features of bradykinesia, for instance, the significant reduction in the amplitude of contractile muscles and the number of repetitions during impact exercise.

### 2.2. Dual-system goal-directed and habitual control in the basal ganglia

*In vivo* microelectrode stimulation and functional imaging studies in monkeys and humans have shown that the output signals of the basal ganglia nuclei are highly consistent with the functional inputs they receive (27). This implies that the basal ganglia can be classified into three distinct neural functional networks, namely limbic, associative, and sensorimotor networks. The phenomenon of early operant conditioning has been effectively used to demonstrate the significant association between a stimulus and its corresponding response (28). However, research conducted over the past two decades has uncovered the ability of animals to encode a causal relationship between their actions and the outcomes they experience. As a result, when behavior is driven by specific goals, the choice of action becomes largely determined by the anticipated utility of the predicted results, leading to potential variations in the effects of different behaviors. To ascertain whether a particular behavior is goal-oriented or habitual, a series of behavioral tests has been developed and employed as an evaluation tool (27, 28).

Yin et al. (29, 30) inactivated the DMS by neurotoxic exposure or local injection of muscimol (a γ-aminobutyric acid agonist). In these animals, behavioral responses were insensitive to outcome devaluation, and goal-directed control was blocked. Consistently, control animals showed induced habitual responding after overtraining, whereas rats with dorsolateral striatum (DLS) lesions failed to establish the habitual behaviors and returned to goal-directed control (30). These studies showed that the DMS and DLS regions are critical to goal-directed behavior and habitual control. Optogenetic studies in rodents demonstrated that DMS and DLS receive fiber tracts from the PFC and the sensory motor cortex (SMC), respectively, to construct goal-directed and habitual control pathways, which participate in motor skill learning and promote action automation (31).

Fitts and Posner (32) proposed that the acquisition of motor skills occurs over three stages: the cognitive stage, the stimulus–response associative stage, and the habitual/autonomous stage. The PFC-DMS pathway is mainly activated at the cognitive stage; the stimulus–response associative stage is a transitional stage from goal-directed control to habitual control that is dominated by the SMC-DLS pathway control; at the habitual stage, the procedure is automated, and can be completed by activating the SMC-DLS pathway, and this stage usually requires relatively fewer cognitive resources (33). Motor skill acquisition involves a transition from goal-directed to habituation control. The condition for achieving autonomous motion execution is overtraining, and the two systems can cooperate and switch according to the behavioral responses.

Goal-directed behavior and habitual control have the same neural basis in human, nonhuman primate, and rodent brains. Studies have found that the human PFC and caudate have high blood oxygen levels during goal-directed behaviors, such as postural transitions, behavioral execution, and cognitive manipulation (34, 35); long-term exercise training promotes the transition from goal-directed control to habitual control, and the activated brain region also transitions from the DMS to the DLS (27, 36). Anatomic and imaging studies have found that other brain regions related to goal-directed control in the human brain include the ventromedial prefrontal cortex (vmPFC), orbitofrontal cortex, and anterior cingulate cortex, while the putamen and supplementary motor area are closely related to habitual control (37).

In addition to the increasing difficulties with the automatic, habitual components of behavior, patients with Parkinson’s disease often experience cognitive dysfunction, which can also appear early during the disease (1). A randomized controlled study found that even though the loss of dopaminergic neurons alters the basal ganglia habitual control, the expression of habitual motor behaviors is still possible in PD by using the executive volitional component of action (2). Despite the presence of deficits of executive functions and implicit learning, the PFC is crucial for this purpose, and individuals with PD can benefit from using their residual executive resources. Therefore, external stimuli and specific techniques exploiting cognitive strategies have been properly developed in order to activate the volitional-executive motor control system and bypass the dysfunctional, habitual, sensorimotor basal ganglia network in patients with PD (4, 17).

## 3. Dysfunction of the basal ganglia circuits and PD

The dopaminergic neurodegeneration within nigrostriatal systems is regionally restricted. Positron emission tomography scans show that dopaminergic depletion mainly occurs in the caudal part of the striatum (23), and autopsy results also confirmed that dopaminergic deprivation is the most severe in the caudal putamen of patients with PD (38). A unilateral rat model of PD was established by intracerebral injection of 6-hydroxydopamine, and the behaviors of PD rats still showed goal-directed control after excessive training, indicating the difficulty in establishing habitual control after repeated exercise (24). Patients with PD show difficulties in learning and execution (or selection) of habitual behaviors initially. With the progressive loss of DA, patients with PD had to rely on the slower, serial goal-directed control system, which consumes more cognitive effort and causes interference with the original target tasks. This may explain why the movements of patients with PD are usually slow in daily life (39).

The goal directed and habitual systems are the neuroscientific basis of dual behavioral control, and impairments of sensorimotor striatum will first hinder the performance of automatic behaviors. Therefore, early in the evolution of PD, many of the movement defects originate from reduced automatic performance such as gait speed and balance, but these abilities can be restored by training with external cues (40). Even patients with PD perform autonomous movements with reduced frequency, amplitude, and speed. In patients with PD, automation does not completely disappear, and this remnant automation may be attributable to the functioning of residual DA in the striatum. Additionally, sensory events (such as visual and auditory cues) have a priming effect on both systems, and external cues may simultaneously activate both systems to execute habits (24, 41).

Difficulty in initiation and execution of movements is also a common movement disorder in patients with PD, and its pathological mechanism has been partially elucidated. From the perspective of the anatomical structure of neural circuits, the goal-directed and habitual control circuits converge to the thalamocortical loop (42, 43). Abnormal basal ganglia outputs have been identified in both patients with PD and animal models, typically with increased neuronal firing rates and abnormal synchronous oscillatory patterns in the cortico-basal ganglia circuits (44, 45). Neural oscillations have been implicated as a novel biological indicator in different neurodegenerative diseases (46, 47). Imaging studies show that the activity of the SMC-DMS pathway is increased with reduced efficiency in patients with PD; at the transition stage of skill acquisition from goal-oriented to habitual control in patients with PD, the functional connectivity of the PFC-DLS pathway is not lower than that in healthy controls (48–50). These results suggest that after striatal dopaminergic denervation, signals from the goal-directed system need to antagonize the strong inhibitory effect of the habitual control system to perform the action effectively. This point of view may also explain many of the characteristics of bradykinesia, such as the significant reduction in the amplitude of contractile muscles and the number of repetitions during impact exercise.

Surgical destruction of the corresponding basal ganglia nuclei (such as STN or GPi) can slow the progression of PD (51). The underlying mechanism may be related to the reduction in the strong inhibitory oscillatory inputs of the habitual pathway, which reduces the burden of action execution without inducing new motor disorders (51). In the dual-system model of goal-directed and habitual control, deep brain stimulation can significantly improve the motor symptoms in patients with PD. Deep brain stimulation plays a therapeutic role by suppressing the inhibitory inputs of the habitual pathway through high-frequency stimulation, and any therapy that stops or reduces the disordered signals in the stimulus–response habitual control circuit is postulated to facilitate the implementation of goal-directed control (52).

## 4. Exercise and PD

### 4.1. Therapeutic significance of physical exercise in clinical rehabilitation of PD

Exercise is a form of physical activity that can be modified in terms of form, structure, and repetitions to adapt to the needs of any part of the body. As a non-pharmaceutical therapy, exercise-induced neuroplasticity facilitates improvement of cognitive functions and motor performance in healthy individuals, older adults, and patients with neurodegenerative conditions (53, 54). Treadmill training is effective for improving mobility and cognition in patients with PD (55, 56). Although studies demonstrated that treadmill intervention contributes to restoring habitual behaviors such as gait, evidence for the plasticity of the control functional regions in the brain in patients with PD remains elusive. Fisher et al. (57) demonstrated that treadmill intervention consisting of three 1 h/week treadmill sessions for 8 weeks could upregulate the expression of D2 receptors in the basal ganglia of patients with early-stage and non-medicated PD. Similarly, adults who engage in regular physical activity show less age-related loss of DA compared with less active adults (58). Using a resting state functional magnetic resonance imaging design, Jarrahi et al. (59) highlighted that high exercise-related cardiorespiratory fitness is associated with increased within network connectivity in cognitive networks known to be impaired in PD, and the physical performance test shows a similar trend. While exercise-mediated restoration of cognitive and motor control-related regions has been shown in patients with PD, there remains a significant gap in knowledge in understanding the role of that exercise training may play in ameliorating the imbalance between the goal-directed and habitual control systems in patients with PD. In addition, treadmill exercise could improve the Unified Parkinson’s Disease Rating Scale score and the quality of life of patients with PD, and this rehabilitative effect persists for some time after training is stopped (60). However, from the perspective of goal-directed control, treadmill exercise is regarded as an external cue that exerts a compensatory effect on the internal defect pathway of patients with PD. Whether the goal-directed control circuit is involved in the behavioral improvement is unknown, and the related rehabilitative mechanism remains to be further elucidated.

In the pathological state of PD, although DA depletion leads to cognitive decline and patients eventually develop dementia, patients in the early and middle stages of the disease still retain explicit learning (43). Therefore, motor cognitive neuro-rehabilitation strategies have been developed to activate the cognitive motor control system and bypass the dysfunctional, habitual, sensorimotor basal ganglia network in PD (42). For patients with early- and mid-stage PD, some language or proprioceptive training can be used to activate cognitive engagement, and interval training can also be used to improve attention or strengthen training motivation (61). Treadmill exercise combined with cognitive intervention (motor-cognitive rehabilitation) has been confirmed to significantly improve gait, including speed, step length, postural stability, gait rhythm, and consistency, in patients with early PD (62). Most studies have shown that motor-cognitive rehabilitation therapy can improve the motor function in patients with PD, but some studies have yielded inconsistent findings (63, 64). This inconsistency may be attributable to the differences in the proportion of feedback and cognitive engagement during treadmill training. Moreover, late in PD, cognitive impairment and dementia symptoms disturb rehabilitation. Silveira et al. (65) compared the differential effects of aerobic exercise and goal-directed exercise protocols on patients with advanced PD (with cognitive impairment). Their results showed that aerobic exercise had a more significant effect than goal-directed exercise on the improvement of executive function in healthy individuals and patients with PD. Further studies showed that aerobic exercise enhances frontal and parietal response inhibition in healthy older adults. These findings suggest that exercise-induced plasticity in the frontal lobe may prevent the defects of habitual motor control in patients with PD by regulating attention processes. In summary, the neuro rehabilitation of motor-cognitive therapy is sensitive to the parameter set for the exercise plan, and the formulation of a rehabilitation strategy should fully consider the stage of the patient’s PD, parameters of the exercise, and other individualized characteristics. Exploration of the mechanisms of motor -cognitive rehabilitation can be helpful in providing more precise exercise parameters for clinical rehabilitation and for developing a variety of combined exercise interventions.

Numerous investigators have formerly suggested schedules intended to improve particular motor constituents of PD, encompassing power, movement, stride, joint mobility, muscle elongation, body position, stability, and stamina (66). Amalgamating motor and cognitive methodologies, such as the treadmill, virtual reality, and Cueing techniques, can lead to the most favorable outcomes. Studies have found that following a goal-based aerobic intensive rehabilitation program has a positive effect on improving motor function and reaction time (67). These results explain that the role of attention executive components is critical, especially when patients with PD are trained with cues and feedback. In a double-blind, randomized controlled trial, Van der Kolk et al. (68) used virtual reality software and real video (Participants exercised with remote supervision on a stationary cycle. Exergaming was used to keep participants motivated to complete the training) to provide an effective aerobic exercise training method and Movement Disorders Society—Unified Parkinson’s Disease Rating Scale (MDS-UPDRS) to assess exercise performance. MDS-UPDRS motor score in the “off” state showed a significant difference between the aerobic exercise group and the control group, suggesting that aerobic exercise intervention can improve motor function scores in patients with Parkinson’s disease. Finally, a study involving 26 patients with basal ganglia degeneration examined the effects of a long-term comprehensive rehabilitation treatment program (69). The results indicated that apparently divergent findings on basal ganglia contribution to social decision-making may instead reflect a model where higher-order learning processes are dissociable from trial-and-error learning, and can be preserved despite basal ganglia damage.

### 4.2. Exercise-enhanced neuroplasticity in PD

Exercise improves cognitive and motor function by inducing molecular, cellular, and systems level plasticity. The intensity, form, density, challenge, and other parameters of exercise are the key factors affecting neuroplasticity in brain injury or neurodegenerative diseases. Exercise-induced neuroplasticity for the prevention and treatment of PD includes the regulation of synaptic conduction in the DA and non-DA systems and the reorganization of functional brain networks.

Exercise regulates DA neurotransmission in patients with PD. Moderate-intensity exercise can significantly improve the efficiency of mitochondrial electron transport, increase adenosine triphosphate synthesis, and inhibit the apoptosis of DAergic neurons in the substantia nigra (70). Previous researchers believe that the antioxidative stress and neuroprotective effects of exercise intervention on the DA system are affected by the timing of the exercise intervention (71, 72). The earlier the exercise intervention and the milder the disease, the more significant the antioxidative stress effect (70). The possible mechanisms include exercise-induced reduction in the oxidative stress response in dopaminergic neurons and increased expression of tyrosine hydroxylase and dopamine transporter, which promotes DA synthesis and accelerates DA transmission, resulting in a neuroprotective effect (71).

Exercise-induced neuroplasticity may also be achieved through regulation of non-DAergic neurotransmission systems such as corticostriatal glutamatergic synaptic transmission. The plasticity and synaptic excitability mediated by glutamate and its receptors are the basis of action learning and execution. After striatal dopaminergic denervation, exercise can regulate the expression of receptors such as metabotropic glutamate receptors, N-methyl-D-aspartic acid receptors, α-amino-3-hydroxy-5-methyl-4-isoxazole-propionic acid receptors, and the cannabinoid receptor 1 at corticostriatal synapses in PD models (73, 74). Exercise can also inhibit the excessive release of presynaptic glutamate and postsynaptic excitotoxicity and reduce the excessive stimulation of glutamate to remodel information transmission in the corticostriatal pathway (75, 76).

Neurotrophins are also sensitive targets of exercise regulation. Exercise can promote the expression of brain derived neurotrophic factor and glial cell line-derived neurotrophic factor both in patients with PD and animal models, providing nutritional support for neuronal survival, growth, and synaptic plasticity (77–79). Fontanesi et al. (80) found that 4 weeks of comprehensive rehabilitation training (combined functional, aerobic, and goal-directed training) improved motor and non-motor symptoms in patients with PD at clinical stage 2–3, and significantly upregulated brain derived neurotrophic factor-TrkB signal transduction; further analysis showed that the Unified Parkinson’s Disease Rating Scale scores of patients with PD were significantly positively correlated with upregulation of the TrkB protein, suggesting that exercise may slow disease progression by this mechanism.

Exercise-induced plasticity can also promote the reorganization of brain functional areas. Duchesne et al. (81) found functional changes in motor learning related brain structures (including the hippocampus, striatum, and cerebellum) that are consistent with improved behavioral performance after 12 weeks of aerobic exercise in patients with PD. Studies using invasive electrophysiological techniques have shown that exercise can regulate the time-frequency phase-synchronization between spikes and local field potentials in PD animal models and suppress the abnormal oscillatory signals of SMC-DLS, thereby remodeling the corticostriatal functional circuits (59, 82, 83). Exercise is critical for the development of plasticity in the motor-cognitive control brain regions. At present, only a few studies have evaluated the effects of exercise on the plasticity of the cortical-basal ganglia functional networks, with no definite evidence to elucidate that exercise regulates the functional remodeling of goal-directed and habitual control systems. The results of such studies may provide an important breakthrough for the development of precise and individualized exercise plans.

## 5. Discussion

Overall, motor skill training or aerobic exercise are effective rehabilitation methods for patients with PD. Exercise-induced neuroplasticity can improve cognitive and behavioral performance; moreover, exercise intervention mediates the remodeling of neural structure and function that may promote the restoration of habitual control in patients with PD. Animal and human studies have provided the means to evaluate exercise rehabilitation at the micro and macro levels. The combination of animal and human measurements with parallel experimental designs is an important breakthrough in elucidating the neurobiological basis of exercise-induced neuroplasticity and will facilitate assessments of the influence of physical exercise on motor and cognitive functions and comprehensive and precise management of PD through feedback for the results.

This article reviews the role of the dual system in the occurrence and development of PD as well as the neuroplastic mechanisms of exercise in improving PD. However, a limitation of this study is that it is a narrative review. This may limit us to simply describing the research progress on this topic, but may allow future validation of the effects of exercise on clinical symptoms. Indeed, the evidence of goal orientation of motion regulation and functional remodeling of the habit control system has been overlooked and should be a focus of future research.

## Author contributions

TZ: Writing – original draft. KS: Supervision, Writing – Review & Editing. WC: Writing – review & editing.
